# Topiramate-Induced Lithium Toxicity

**DOI:** 10.7759/cureus.1804

**Published:** 2017-10-26

**Authors:** Afaque H Khan, Shazia Q Shah

**Affiliations:** 1 Psychiatry, Heartland Behavioral Healthcare, Department of Mental Health and Addiction, State of Ohio, Northeast Ohio Medical University; 2 Public Health, Uthealth University of Texas Health Science Center at Houston/utsw (dallas)

**Keywords:** compulsive urge of food intake, topiramate, lithium toxicitiy, binge eating disorder, schizoaffective disorder bipolar type 1

## Abstract

This case report discusses a suspected case of lithium toxicity following the administration of topiramate (TPM). Our patient is a 47-year-old man who has been an inpatient for the past year and was diagnosed with schizoaffective disorder bipolar type 1 for the past 20 years according to the criteria of the Diagnostic and Statistical Manual of Mental Disorders, fifth edition (DSM-5). He manifested symptoms of lithium toxicity following administration of TPM to ameliorate the compulsive consumption of food and liquids. The patient was already being treated with lithium carbonate for mania, labile mood, and aggressive behavior for the past year. The patient developed symptoms of lithium toxicity on the fourth day of the TPM treatment. We transferred the patient to the emergency department where he was diagnosed and treated for renal insufficiency due to lithium toxicity. After halting the TPM and reintroducing lithium carbonate, the patient’s laboratory results improved. This case illustrates the potential toxic interaction of medications with a narrow therapeutic index like lithium.

## Introduction

Numerous studies have shown the mechanism of action of topiramate (TPM) has a wide spectrum of pharmacological properties. TPM has been used as a mood stabilizer in patients with bipolar and schizoaffective disorders and has been used to treat anorexia, bulimia, epilepsy, migraines, essential tremors, and cluster headaches. It is also effective in treatment-resistant bipolar disorder by augmenting the effects of lithium. Recent literature has also documented TPM use in treating binge eating disorder (BED) and as an aid for weight loss [1]. TPM reduces the frequency of the voltage-sensitive sodium channels, which plays a key role in the treatment of epilepsy. TPM potentiates the inhibitory effects of gamma-aminobutyric acid-(GABA)-A in the brain and enhances the effects of GABA-stimulated chloride influx in the cerebellar and cortical neurons, increasing the frequency of activation of the GABA-A receptor in the brain, and exhibits anticonvulsant action. TPM also inhibits the excitatory pathways of α-amino-3-hydroxy-5-methyl-4-isoxazolepropionic acid (AMPA) and glutamate receptors. TPM inhibits high-voltage activated calcium channels and reduces their neurotransmitter release, inhibiting the calcium-dependent second messenger system. TPM also has an inhibitory action of carbonic anhydrase at the proximal tubular level. TPM has been considered a weak carbonic anhydrase (CA) inhibitor, which offers no therapeutic value in the treatment of epilepsy and other conditions, but does determine some of its side effects, such as hyponatremia, metabolic acidosis, and increased risk of nephrolithiasis [2].

## Case presentation

A 47-year-old African American man was admitted to the psychiatric state hospital from a group home, as he had become increasingly aggressive, violent, and threatening toward other residents and staff. He became noncompliant with his treatment prior to this relapse. Upon admission to the hospital, the patient was hyperverbal and presented with pressured speech and flight of ideas. He had manifested aggressive, threatening behavior, and insomnia for the past seven days. The patient was started on lithium carbonate to stabilize his mood and paliperidone to ameliorate his symptoms of mania and psychosis. The results of his comprehensive metabolic panel, thyroid panel, complete blood chemistry, and HbA1c tests were within reference ranges. He responded rapidly to the above combination and stabilized. His schizoaffective bipolar type 1 condition was in remission. We monitored his lithium levels weekly with a comprehensive metabolic panel (CMP) and thyroid panel every six months. During hospitalization, the patient developed a compulsive urge to drink an excessive amount of fluids which was managed by daily weight monitoring and water restriction. For the past eight months, the patient developed a compulsive urge to eat excessive amounts of food and had a rapid gain of 40 lbs. Despite various trials of behavioral modifications and therapies, we were unable to control above this compulsive consumption behavior. The patient urgently needed pharmacological intervention to prevent further weight gain, comorbidities, and metabolic syndrome. He was started on TPM for mood stabilization and compulsive excessive food intake. His lithium levels, HBA1c, thyroid panels, CMP, and complete blood count (CBC) all were within reference ranges prior to the TPM administration (Table 1).

**Table 1:** Laboratory test results with lithium carbonate prior to TPM administration for compulsive excessive food intake NA: not applicable; TPM: topiramate

Analyte	Patient Values	Reference Range
Lithium	0.47 mEq/L	0.4-1.3 mEq/L
Sodium	141 mEq/L	135-145 mEq/L
Creatinine	1.1 mg/dL	0.50-1.20 mg/dL
Blood Urea Nitrogen	15 mg/dL	8.0-22.0 mg/dL
Creatinine/Blood Urea Nitrogen Ratio	17	10.0-22.0
Weight Change	0 lbs	NA
Potassium	3.8 mEq/L	3.5-5.0 mEq/L

His kidneys were functioning within reference levels. On the fourth day of treatment, the patient appeared disoriented, increasingly confused, and delirious. He had slurred speech, tremors in both hands, and an unsteady gait. He presented with rapid and shallow breathing. He had concerns of nausea, vomiting, inability to eat, and increased urinary frequency. We suspected lithium toxicity, and treatment with TPM and lithium carbonate were stopped immediately. We ordered an immediate evaluation of laboratory values including CMP, lithium level, thyroid panel, and CBC. His lithium levels were in the toxic range; his creatinine and blood urea nitrogen (BUN) were above the reference ranges (Table 2).

**Table 2:** Laboratory test results following TPM administration for compulsive excessive food intake NA: not applicable; TPM: topiramate

Analyte	Patient Values	Reference Range
Lithium level	1.46 mEq/L	0.4-1.3 mEq/L
Sodium	131 mEq/L	135-145 mEq/L
Creatinine	3.4 mg/dL	0.50-1.20 mg/dL
Blood Urea Nitrogen	47 mg/dL	8.0-22.0 mg/dL
Creatinine/Blood Urea Nitrogen Ratio	23	10.0-22.0
Weight Change	-6 lbs	NA
Potassium	2.9 mEq/L	3.5-5.0 mEq/L

He was transferred to the emergency department where he was diagnosed and treated for renal insufficiency secondary to lithium toxicity. Later, the patient showed significant improvement with conservative and supportive treatment, including rehydration and correction of electrolytes imbalance. The results from his final laboratory assessment are presented in Table 3.

**Table 3:** Laboratory test results following halted TPM treatment and reintroduction of lithium carbonate regimen NA: not applicable; TPM: topiramate

Analyte	Patient Values	Reference Range
Lithium level	0.79 mEq/L	0.4-1.3 mEq/L
Sodium	141 mEq/L	135-145 mEq/L
Creatinine	1.1 mg/dL	0.50-1.20 mg/dL
Blood Urea Nitrogen	15 mg/dL	8.0-22.0 mg/dL
Creatinine/Blood Urea Nitrogen Ratio	17	10.0-22.0
Weight Change	0 lbs	NA
Potassium	3.8 mEq/L	3.5-5.0 mEq/L

## Discussion

Weight gain and BED are ongoing problems associated with psychotropic medications. Management of these associated conditions is both hectic and time-consuming. After the failure of behavioral and lifestyle modifications, physicians have little choice but to utilize pharmacological interventions. TPM is a useful mood stabilizer in addition to its recent use in affecting weight loss. Currently, no psychotropic medications available include efficacy for weight loss. Multiple case studies indicate long-term effectiveness and tolerability of topiramate in patients with a binge eating disorder and obesity. Treatment with TPM has shown improvement in patients with symptoms of binge eating disorder and obesity [3]. In a randomized controlled trial, TPM was found effective and well tolerated in the short-term treatment of binge eating disorder with obesity. A small number of patients presented with side effects of headache and paresthesias [4]. Our patient had been on lithium for the past year without any significant adverse effects. TPM can increase plasma lithium levels significantly, by five-fold [5]. He developed lithium toxicity due to the pharmacokinetic interaction of TPM with lithium, affecting renal excretion. TPM may increase the lithium level by increasing the excretion of sodium. The potential mechanisms of TPM-induced lithium toxicity appear to be both pharmacokinetic (i.e., the competition for renal excretion) and pharmacodynamic (i.e., the weight-loss-induced decrease in sodium-lithium countertransport activity). TPM also reportedly increases plasma lithium levels by 140%, along with increasing plasma lithium clearance by 36%, decreasing the area under the curve by 12%. Both effects are due to TPM's inhibitory action on carbonic anhydrase in the proximal tubule of the kidneys, and this consequently affects lithium renal clearance [2].

## Conclusions

As healthcare providers, we do not recommend TPM for use as a weight-loss aid, but we also need to prevent patients from developing conditions like coronary heart disease, diabetes, hypertension, and metabolic syndrome—conditions that often develop due to the drastic weight gain associated with psychotropic medications. Unfortunately, our patient developed lithium toxicity due to the pharmacokinetic interaction between TPM and lithium. Our patient made a rapid recovery with conservative treatment, including rehydration and correcting his electrolyte imbalance. Behavioral modifications were reinitiated for the binge eating disorder since TPM is not recommended in this case. More research is required to explore a therapeutic approach for patients with binge eating disorders. Our case report underscores the importance of psychopharmacology; psychiatrists should be aware of the potentially hazardous interactions of psychotropic medications with a narrow therapeutic index such as lithium.
